# Patient-reported clinical characteristics and healthcare utilization in Spinal Muscular Atrophy (SMA): a cross-sectional study from Iran

**DOI:** 10.1186/s13023-026-04366-7

**Published:** 2026-05-11

**Authors:** Bita Montazeri, Rajabali Daroudi, Ali Darvishi, Morteza Heidari, Majid Anabi, Ramin Heshmat

**Affiliations:** 1 Department of Pharmacoeconomics, Faculty of Pharmacy, Tehran Medical Sciences, Islamic Azad University, Tehran, Iran; 2 Department of Health Management, Policy and Economics, School of Public Health, Tehran University of Medical Sciences, Tehran, Iran; 3 Workplace Health Promotion Research Center, Research Institute for Health Sciences and Environment, Shahid Beheshti University of Medical Sciences, Tehran, Iran; 4 Department of Health Policy and Management, School of Public Health and Safety, Shahid Beheshti University of Medical Sciences, Tehran, Iran; 5 Department of Pediatrics, Children’s Medical Center, Tehran University of Medical Sciences (TUMS), Tehran, Iran; 6 Chronic Disease Research Center (CDRC), Endocrinology Metabolism Research Institute (EMRI), Tehran University of Medical Sciences (TUMS), Tehran, Iran

**Keywords:** Spinal muscular atrophy, Patient reported outcome measures, Health care costs, Patient reported data, Iran

## Abstract

**Background:**

Spinal Muscular Atrophy (SMA) is a rare, inherited neuromuscular disorder characterized by progressive muscle wasting and imposing a significant clinical and economic burden on both patients and health care systems. Although global research on SMA is expanding, limited evidence is available from low- and middle-income countries, including Iran. This study aimed to provide a comprehensive profile of patients with SMA in Iran using self-reported data, with a focus on clinical characteristics, economic burden, and patterns of healthcare utilization.

**Methods:**

A cross-sectional study was conducted among 73 patients with SMA identified through the national SMA patient advocacy association. Data were collected via structured telephone interviews using a pre-tested questionnaire covering seven domains: sociodemographic characteristics, clinical profile, and health care utilization as prescribed by physicians, direct medical and non-medical costs. Descriptive analysis was performed using SPSS (v23).

**Results:**

Among the 73 participants (49.3% female), the majority had SMA Type 2 (63.0%) and were older than 10 years (97.3%). Hospitalization was reported by 52.1% of patients, with SMA-related admissions incurring significantly higher costs (average costs: $240.764) and longer stays. Regarding disease-modifying therapy, 56.2% received SMN-targeted treatments. Perceived efficacy assessments revealed that 72% and 81.25% of Nusinersen and Risdiplam -treated patients reported “good or significant” clinical improvement, respectively. Outpatient service use was variable: 49.3% had never received occupational therapy, and 74.0% had not engaged in physiotherapy. General practitioner (GP) visits were more common (68.5%), while consultations with pulmonologist and cardiologist visits were infrequent (24.7% and 27.4%, respectively). Only 19.2% reported having annual preventive check-ups.

**Conclusion:**

The findings underscore considerable heterogeneity in service utilization and economic burden among patients with SMA in Iran. the underuse of rehabilitation and specialist care may reflect access barriers or financial constraints. There is a critical need for national strategies to improve early diagnosis, ensure equitable access to therapies, and integration of SMA care into existing health insurance frameworks.

## Introduction

Spinal muscular atrophy (SMA) is a rare genetic disorder that significantly impairs quality of life and has attracted the attention of health policymakers worldwide due to its substantial burden [1]. It is recognized as the leading genetic cause of death in infants and children and the second most common autosomal recessive disorder [2]. As of 2010, the global incidence of SMA was estimated at approximately 1 in 10,000 live births, with prevalence in 2017 ranging between 1 and 2 per 100,000 individuals [3, 4].

SMA is a progressive neurodegenerative disease categorized by the degeneration of motor neurons in the brainstem and anterior horn of the spinal cord, both essential for voluntary muscle control [5–7]. Among autosomal recessive disorders, it is second only to cystic fibrosis of mortality [8]. The disease is categorised into subtypes based on severity, with Type 1 being the most severe. Type 1 accounts for approximately 60% of all SMA cases and is the leading genetic cause of infant mortality. Without prompt respiratory support, most infants with Type 1 SMA do not survive beyond two years of age [9].

The direct medical costs associated with SMA are exceptionally high and vary significantly by disease subtype and country. For example, the annual medical cost per patient ranges from $3,320 for Type 3 SMA in Italy [10] to $324,410 for Type 1 SMA in the United States [11]. A 2022 study in Hong Kong estimated the average annual direct medical cost of SMA at $935,570, with annual costs of $2,393,250, $413,165, and $40,735 for Types 1, 2, and 3, respectively [9]. Additionally, average annual non-medical direct costs in 2018 U.S. dollars were reported as $25,880 in Spain [12], $136,800 in Germany [13], $9,440 in another German study [14], and $74,910 for Type 1 patients in Sweden [15].

The burden of SMA extends well beyond its clinical consequences, imposing significant social and economic challenges_ particularly in high-income countries where, its societal costs are considerable. In Spain, for example, the estimated social costs of SMA exceed those of other chronic conditions such as stroke (€27,711 per patient in 2012), symptomatic chronic heart failure (€12,995 - €18,220 per patient in 2012), and AIDS (€17,300 per patient in 2010) [16].

Although comprehensive national data on the distribution of SMA subtypes in Iran are limited, preliminary figures from local registries provide some insight. Between October 2021 and September 2022, 59 SMA patients were registered at a selected hospital in Iran; 52.5% were female, with a mean age of 4.08 ± 4.98 years. Among these, 47% had Type 1, 29% had Type 2, and 24% had Type 3. 19% of the patients had died, all of whom had Type 1 SMA [17]. Another report from the Iranian SMA Registry, published in 2022, analyzed data from 781 SMA patients under 18 years of age. Of these, 164 had died—primarily those with Type 1 SMA, with a median survival time of 23 months. Among the 617 surviving patients, the rate of consanguinity was 52.4%, while only 24.8% had a positive family history of SMA. Type 3 was the most common subtype among the surviving patients [18]. Previous studies have primarily focused on the clinical characteristics of patients at the time of hospital referral, without following them after discharge. However, SMA is a chronic condition that requires ongoing supportive care. When patients do not receive the services recommended by clinical guidelines, their quality of life is likely to decline, and healthcare system costs are likely to increase. Therefore, examining patients’ utilization of health services can provide valuable insights for policymakers seeking to address the needs of individuals with SMA. The present study aimed to describe the clinical characteristics of patients with SMA in Iran and examine their patterns of healthcare utilization.

## Methods

This cross-sectional study aimed to examine the clinical characteristics and healthcare utilization patterns among patients with SMA in Iran. Costs including direct medical and non-medical and indirect costs were also calculated. The study was conducted in 2024 (from 1 May to 29 June) following receipt of ethical approval from the relevant ethics committee.

### Participants and sampling

Given the limited data available on SMA patients in Iran as a rare disease population, convenience sampling was used. Participants were recruited through the Iranian SMA Patient Advocacy Association. All registered members (120 SMA patients) were contacted by telephone, and 73 agreed to participate and were included in the study.

### Data collection

Due to the geographical dispersion of patients with SMA across the country and their mobility limitations, all data were collected via telephone interviews from the research office at a referral hospital for SMA patients. The first author, a health economist with prior training and experience in conducting structured interviews for health services research, conducted all interviews. Each interview lasted approximately 30 to 45 min. For patients under 18 years of age, interviews were conducted with their caregivers, primarily parents.

At the beginning of each interview, the researcher explained the study’s objectives, assured confidentiality, and clarified that participants were not required to disclose their identity. Participants were informed that they were free to withdraw at any time and were encouraged to share their experiences openly. All participants provided informed consent before inclusion in the study.

### Questionnaire

Data were collected using a pre-tested structured questionnaire covering six main sections:

1. **Demographic and socio-economic characteristics** (e.g. age, sex, education, insurance type, parental occupation).
2. **Disease-related clinical characteristics** (e.g., SMA type, age of onset, comorbidities).
3. **Healthcare utilization and reasons for refusing needed care**
4. **Costs,** including direct medical costs (e.g. medications, specialist visits, rehabilitation services as prescribed by physicians, and associated costs), direct non-medical and indirect costs (e.g. home care, transportation, assistive devices, caregiver absenteeism)
5. **New medicine usage patterns and perceived effectiveness**
6. **Family background and caregiving context**

The interview began with demographic questions, followed by open-ended questions related to the research objectives, including:

- Age at diagnosis
- Diagnostic test modality and associated costs
- Family history of SMA among first-degree relatives
- Presence of comorbidities
- Functional status (e.g., ability to sit or walk independently)
- Hospitalization or surgery history related to SMA and total costs
- Medications and services used as prescribed by physicians
- Reasons for discontinuation of health services, if applicable

Reasons for not receiving prescribed health services were also recorded. The full questionnaire is available from the corresponding author upon reasonable request.

### Data management and analysis

Responses were recorded manually during the telephone interviews using the structured questionnaire. The first author entered all data into Microsoft Excel. Data were then exported to SPSS software (version 23) for analysis.

Descriptive statistics were used to summarize patient characteristics, disease profiles, and cost components. For categorical variables (e.g. gender, education, and insurance status), frequencies and percentages were reported. For continuous variables (e.g. costs), measures of central tendency and dispersion _including mean and standard deviation_ were calculated.

Healthcare service utilization was assessed as the number of services used by patients over the past year. The difference between actual services received and those prescribed by physicians was then calculated. No inferential statistical tests were applied due to the descriptive nature of the study.

All medical costs were adjusted to present value (2025) based on the Relative Value Unit (RVU) system in Iran. Other cost items were similarly adjusted. To facilitate international comparison, all cost values were converted to US dollars using the average exchange rate at the time of data collection (1 USD = 430,000 Iranian Rails) [19].

### Ethical considerations

The Ethics Committee of Azad University of medical science (Code: IR.IAU.PS.REC.1402.525) approved the study protocol. All participants were informed of the study’s voluntary nature, and verbal informed consent was obtained before data collection. Collected data were anonymized and stored securely to ensure confidentiality.

## Results

Table 1 presents the sociodemographic and clinical characteristics of the 73 individuals with SMA included in the study. The gender distribution was nearly equal, with 50.7% males. The majority of participants (97.3%) were older than 10 years, with the highest proportion (49.3%) in the 10–20 years age group. Regarding educational attainment, over half of the participants (53.4%) had completed secondary or high school education, and 30.1% held a university degree. Regarding health insurance coverage, 94.5% of participants were covered, while only 5.5% had no basic health insurance. Notably, the majority (69.9%) lacked supplementary health insurance coverage. The most common age at diagnosis was early childhood (42.46%), followed by late childhood or later (21.92%). Genetic testing and combined diagnostic methods were the primary modalities used, with 56.16% of cases diagnosed through a combination of tests. The most prevalent form was SMA type 2, accounting for 63.01% of cases, and 45.21% of participants reported a positive family history of the disease. Only 15.07% presented with comorbid conditions. Regarding, functional status, 57.53% of individuals were current wheelchair users, while 4.11% required a wheelchair but did not have access to one (Table 1).

**Table 1:** Baseline characteristics of SMA patients

Variables	Sub group	Frequency, number (%)
Gender	Female	36(49.31)
	Male	37(50.69)
Age groups	less than 10 years old	2(2.74)
	10–20 years old	36(49.32)
	> 20 years old	35(47.95)
Education	No formal education or primary education	12(16.44)
	Secondary/high school education	39(53.42)
	University degree	22(30.14)
Age at diagnosis	Prenatal	1(1.37)
	Infancy	10(13.70)
	Early childhood	31(42.46)
	Late childhood or later	16(21.92)
	Adolescence and adulthood	15(20.55)
Diagnostic test Modality	Genetic testing	24(32.88)
	EMG/NCS	8(10.96)
	Combination of different methods	41(56.16)
	SMA type 2	46(63.01)
	SMA type 3	27(36.99)
SMA Family History	No	40(54.79)
	Yes	33(45.21)
Presence of Comorbidities	No	62(84.93)
	Yes	11(15.07)
Wheelchair Use Status	No wheelchair needed	28(38.36)
	A wheelchair is needed, but not available	3(4.11)
	Currently using a wheelchair	42(57.53)
Sitting Ability	No	49 (67.12)
	Yes	24 (32.88)
Basic health insurance coverage	No	4(5.48)
	Social Security Organization	42(57.53)
	Iran Health Insurance Organization	10(13.70)
	other	17(23.29)
Complementary health insurance	No	51(69.86)
	Yes	22(30.14)

According to self-reported data from individuals with SMA, 47.95% of participants reported no history of hospitalization since diagnose. Of the remaining participants, 31.51% had been hospitalized for reasons unrelated to SMA, with an average length of stay of 2.91 days and an average hospitalization cost^1^ of $240.764. A total of 20.55% reported hospitalization related to SMA, with a notably higher mean length of stay (6.8 days) and average cost ($1207.99). Among SMA-related hospitalizations, the most common cause was spinal deformity correction (40%), followed by hospitalizations for diagnostic procedures and pulmonary disorders. The costliest hospitalization types were spinal and genu varum correction surgeries, each exceeding $ 2325.58 per admission (Table 2).

**Table 2:** Self-reported hospitalization history, length of stay, and cost among patients with SMA

Hospitalization-category	Frequency, number (%)	Length of stay		Cost	
		Mean	St. Dev	Mean	St. Dev
No Hospitalization History	35(47.95)	-			
Non-SMA-Related -Hospitalization History	23(31.51)	2.91	3.34	241	530
SMA-Related -Hospitalization History	15(20.55)	6.8	8.2	1,208	1,204
*Spinal Deformity Correction*	6(40.00)	6.16	2.99	2,442	465
*Pulmonary Disorders*	2(13.33)	1.00	0.00	311	415
*Genu Varum Correction*	1(6.67)	3.00	-	2,442	-
*Sensory Disturbance in Extremities*	1(6.67)	30.00	-	-	-
*Hospitalization for diagnosis*	5(33.33)	6.00	8.24	81	124

### The utilization of health services

Table 3 summarizes self-reported medication utilization and perceived therapeutic outcomes among 73 patients with SMA, stratified by disease subtype (Type 2: *n* = 46; Type 3: *n* = 27). Overall, 56.16% (*n* = 41) received SMN-targeted therapies, with Nusinersen (Spinraza) accounting for 60.98% of administered treatments. A notable disparity in therapy distribution was observed: Nusinersen was more prevalent in Type 3 patients (75.00% of treated cases) compared to Type 2 (47.62%), whereas Risdiplam (Evrysdi^®^) was more frequently utilized in Type 2 (52.38% vs. 25.00% in Type 3). Perceived efficacy assessments revealed that 72.00% of Nusinersen-treated patients reported “good or significant” clinical improvement, with higher satisfaction rates in Type 3 (86.67%) compared to Type 2 (50.00%). For Risdiplam, 81.25% of recipients endorsed “good or significant” effectiveness, with no adverse effects reported in Type 3 patients. Concurrent medication use included analgesics (16.44% of the cohort) and nutritional supplements/supportive therapies (53.42%). Regarding concomitant medication use included analgesics, utilized by 16.44% (*n* = 12) of the cohort, while 53.42% (*n* = 39) received nutritional supplements or supportive therapies. Notably, analgesic use was slightly higher in Type 3 (18.52%) than in Type 2 (15.22%), whereas nutritional supplementation was more common in Type 2 (56.52%) compared to Type 3 (48.15%).

**Table 3:** Self-reported medication profiles and perceived efficacy of SMN-targeted therapies in patients with SMA

Variables	Sub groups	Frequency, number (%)		
		Total	SMA type 2	SMA type 3
***SMN-Targeted Therapies***				
Received/Not Received	Not Received	32(43.84)	25(54.35)	7(25.93)
	Received	41(56.16)	21(45.65)	20(74.07)
Type of Received Medicine	Nusinersen (Spinraza)	25(60.98)	10(47.62)	15(75.00)
	Risdiplam (Evrysdi)	16(39.02)	11(52.38)	5(25.00)
Perceived Nusinersen Effectiveness	No Effect	0(0.00)	0(0.00)	0(0.00)
	Mild Effect	0(0.00)	0(0.00)	0(0.00)
	Moderate Effect	7(28.00)	5(50.00)	2(13.33)
	Good or Significant Effect	18(72.00)	5(50.00)	13(86.67)
Perceived Risdiplam Effectiveness	No Effect	1(6.25)	1(9.09)	0(0.00)
	Mild Effect	1(6.25)	1(9.09)	0(0.00)
	Moderate Effect	1(6.25)	0(0.00)	1(20.00)
	Good or Significant Effect	13(81.25)	9(81.82)	4(80.00)
***Analgesics / Painkillers***				
Not Received		61(83.56)	39(84.78)	22(81.48)
Received		12(16.44)	7(15.22)	5(18.52)
***Nutritional Supplements /Supportive Drugs***				
Not Received		34(46.58))	20(43.48)	14(51.85)
Received		39(53.42)	26(56.52)	13(48.15)

Table 4 presents self-reported data on the utilization of occupational therapy, physiotherapy, and medical visits among patients with SMA Types 2 and 3. Most participants reported no history of occupational therapy (49.32%) or physiotherapy (73.97%), although a notable proportion of SMA Type 3 patients were currently engaged in occupational therapy (51.85%). Among those who participated in physiotherapy, the majority reported fewer than 40 sessions annually. The frequency of GP visits indicated that 68.49% of participants had seen a GP at least once in the past year, with 54% reporting 2–4 visits. Pulmonologist consultations were limited, with 75.34% reporting no visits and only 24.66% having at least one. Similarly, cardiologist visits were infrequent, as 72.97% of participants had not visited a cardiologist in the past year. Annual health check-ups were underutilized, with only 19.18% of patients reporting that they had undergone one (Table 4).

**Table 4:** Self-reported ambulatory care utilization in patients with SMA

Variables	Sub groups	Frequency, number (%)		
		Total	SMA type 2	SMA type 3
***Occupational Therapy***				
Occupational Therapy Engagement Status	No history of Occupational Therapy	36(49.32)	25(54.35)	11(40.47)
	Discontinued due to financial barriers	10(13.70)	8(17.39)	2(7.41)
	Currently engaged in occupational therapy	27(36.99)	13(28.26)	14(51.85)
Frequency of Weekly Occupational Therapy Sessions	<=1 session/week	7(25.93)	4(28.57)	3(23.08)
	2 sessions/week	6(22.22)	3(21.43)	3(23.08)
	3 sessions/week	10(37.04)	4(28.57)	4(30.77)
	> 3 sessions/week	4(14.81)	3(21.43)	3(23.08)
***Physiotherapy***				
Physiotherapy Engagement Status	Not engaged	54(73.97)	31(67.39)	23(85.19)
	Engaged	19(26.03)	15(32.61)	4(14.81)
Frequency of Annual Physiotherapy Sessions	< 20 sessions/year	2(10.53)	1(6.67)	1(25.00)
	20–40 sessions/year	11(57.89)	10(66.67)	1(25.00)
	> 40 sessions/year	6(31.58)	4(26.67)	2(50.00)
***General Practitioner Visits***				
General Practitioner Visits Status	Did not visit	23(31.51)	17(36.96)	6(22.22)
	Visited	50(68.49)	29(63.04)	21(77.78)
Annual Number of General Practitioner Visits	1 visit/year	17(34.00)	11(37.93)	6(28.57)
	2–4 visits/year	27(54.00)	17(58.62)	10(47.62)
	> 4 visits/year	6(12.00)	1(3.45)	5(23.81)
***Specialist Consultations***				
Annual Number of Pulmonologist Consultations	0 visits/year	55(75.34)	31(67.39)	24(88.89)
	1 visit/year	18(24.66)	15(32.61)	3(11.11)
Annual Number of Cardiologist Visits	0 visits/year	53(72.60)	30(65.22)	23(85.19)
	1 visit/year	20(27.40)	16(34.78)	4(14.81)
***Check-up***				
Underwent annual check-up	Did Not Have	59(80.82)	37(80.43)	22(81.48)
	Had Annual Check-up	14(19.18)	9(19.57)	5(18.52)

## Discussion

Our study provides a detailed of the clinical profile, sociodemographic, and healthcare utilization patterns among 73 patients with SMA in Iran. Of the total sample, 63% had SMA type 2, and approximately 45% reported a family history of SMA. A previous study by Mansouri et al., examined the clinical characteristics of SMA in Iran using a registry system. Their findings indicated that the majority of patients had SMA type 3, and approximately 24% reported a family history of the disease [18]. The observed differences in findings may be attributed to variations in study samples. While Mansouri et al. focused on SMA patients requiring hospital services, our study followed patients after hospital discharge.

Given the progressive nature of SMA, consistent rehabilitation is crucial for maintaining motor function and preventing complications. Based on a study by Dunaway et al., 86% of SMA patients required physical therapy services, with more than 80% of them receiving theses services in clinical settings [20]. Similarly, Foead et al., emphasized that despite the efficacy of new disease-modifying therapies, patients with SMA continue to require rehabilitation [21]. Therefore, these services should be considered for improving patients’ quality of life. In line with this, our results showed that 57.53% of the participants were current wheelchair users and 4.11% needed a wheelchair but did not have access to one. Furthermore, nearly 50% of patients had never received occupational therapy, and almost 75% did not participate in physiotherapy—highlighting broader concerns regarding the management of chronic neuromuscular disorders. While a notable proportion of SMA Type3 patients were currently engaged in occupational therapy, about 49.32% of participants did not utilize these services, primarily due to financial limitations. Although Iran’s national health insurance system provides support packages for patients with severe illnesses, the scope of coverage is limited, focusing predominantly on hospital expenses. Indirect medical costs, such as wheelchairs and home modifications to improve accessibility, are not included in these packages, placing substantial financial pressure on families [22]. Based on patient reports, additional barriers to care included limited access to specialized service centers, the need for repetitive therapy sessions often requiring caregiver support, and psychological distress leading to discouragement and treatment avoidance. These reasons for non-utilization of services are consistent with findings from the study by Wan et al., which identified financial and social barriers, pragmatic challenges, and a perceived low value of care as key obstacles to service utilization among SMA patients. The authors also suggested that fear and stigma were additional factors, particularly regarding the use of mental health services [23]. Findings from Hanney et al., which assessed barriers to the utilization of physical therapy services, identified logistical constraints and treatment duration were identified as the most significant barriers [24]. These findings align with the results of the present stud, in which several SMA patients reported a lack of access to a nearby clinic. Due to their mobility limitations, frequent use of rehabilitation or physiotherapy services was even more challenging for the patients.

Access to these services is crucial for mitigating disease severity and reducing healthcare costs for both patients and the health system. SMA progression can lead to severe complications, including scoliosis (spinal curvature), joint deformities (e.g., genu varum), respiratory issues (leading to lung infections), and cardiovascular complications requiring hospitalization. Regular medical monitoring and preventative care can delay disease progression and reduce hospital admissions, however, our study found that Iranian SMA Patients only seek medical attention when experiencing pain, neglecting specialist visits and annual check-ups. This pattern suggests a reactive approach to care, which increases medical costs over time, rather than a proactive model focused on early intervention. Our findings indicate that SMA-related procedures—such as spinal deformity corrections—result in significantly longer hospital stays and higher costs compared to non-SMA hospitalizations. These results align with prior research. For example, Tan et al., demonstrated that SMA patients, particularly those with infantile onset, incur substantially higher healthcare costs and resource utilization than non-SMA populations. Their study, which included a broader age group and a matched control cohort, supports our findings regarding the economic burden of SMA-related hospitalizations [11]. Similarly, Sejersen et al. quantified direct medical costs and inpatient stays among Swedish SMA patients, reinforcing the observation that specialized SMA care demands greater hospitalization resources [25]. Additionally, Cardenas et al., compared healthcare resource use in SMA type1 hospitalizations versus non-hospitalized SMA patients, reporting significantly higher costs per admission due to increased procedures, longer stays, and multiple hospitalizations [26].

New technologies can assist in managing disease severity, ultimately enhancing quality of life. Nusinersen and Risdiplam have been introduced as new treatments that support the improvement of SMA patients and have recently become available in Iran. Our findings underscore the high patient-reported efficacy of SMN-targeted therapies, particularly Nusinersen in Type 3 SMA, and highlight variations in treatment patterns across subtypes. Perceived efficacy assessments revealed that 72.00% of Nusinersen-and 81.25% of Risdiplam-treated patients reported “good or significant” clinical improvement. These results align with previous studies demonstrating the critical role of therapy adherence in SMA treatment outcomes. For instance, Kokaliaris et al., found that long-term adherence to Risdiplam significantly improved motor milestone achievement compared to Nusinersen [27]. Similarly, Ashra et al., highlighted differences in treatment efficacy among Iranian SMA patients, emphasizing the need for tailored therapeutic approaches [28]. Kleintjens et al., further underscored the economic consequences of poor adherence, showing that while reduced persistence lowers healthcare costs, it also diminishes health benefits [29]. Based on Yaftian et al., while Nusinersen is highly effective, it remains economically unviable, limiting patient access [30]. In the present study, approximately 56% of patients received new medications to manage disease severity. While these treatments were provided free of charge to patients, their high cost made it difficult for the healthcare system to sustain widespread distribution. As a result, some patients were unable to obtain the medication they needed.

Socio-cultural factors significantly influence healthcare access for SMA patients. Stigma, limited awareness of supplementary therapies, and inadequate advocacy for specialized neuromuscular care often lead to the underutilization of rehabilitative and supportive services. Targeted awareness campaigns aimed at both healthcare professionals and patients could improve service utilization, fostering a more proactive approach to disease management. In comparison with published literature, studies indicate that socio-cultural barriers play a crucial role in healthcare accessibility. Research highlights disparities in access due to social class and economic status, reinforcing the need for targeted interventions. Additionally, cultural awareness has been identified as a key factor in shaping effective healthcare policies [31].

Studies have emphasized that financial constraints and systemic inefficiencies significantly limit access to medical visits for individuals with chronic conditions, including SMA. Similarly, research on healthcare utilization among people with disabilities in Iran has shown that barriers to routine medical care disproportionately affect those with limited financial resources and coverage gaps [32]. This lack of preventive care not only delays the detection of secondary complications but also underscores the urgent need for an integrated SMA care model. Strategies such as decentralized care models, enhanced telemedicine initiatives, and the development of multidisciplinary clinics could serve as promising solutions to bridge these healthcare gaps and improve long-term patient outcomes. Furthermore, for patients with limited financial resources or inadequate supplementary coverage, these expenses likely deter necessary interventions, leading to delayed care or avoidance of treatments that could enhance mobility and overall quality of life. Addressing these financial constraints is essential to ensuring equitable access to SMA therapies. Finally, despite the valuable insights gained from our study, several limitations must be acknowledged. The relatively small sample size and reliance on self-reported data introduce the potential for recall and selection biases. Moreover, while our findings provide regional perspectives, differences in healthcare systems across countries limit the direct comparability and generalizability of results. Additional, variability in diagnostic criteria, treatment protocols, and insurance structures necessitates cautious interpretation when extrapolating findings to broader SMA populations.

## Conclusion

The study reveals a complex interplay of financial constraints, infrastructural limitations, and systemic inefficiencies that curtail SMA patients’ access to vital health services. Addressing these access limitations requires a coordinated response from policymakers, healthcare providers, and patient advocacy groups. Key strategies- including expanding insurance coverage, funding non-medical support, increasing the availability of specialized and rehabilitation services, establishing comprehensive, multidisciplinary care networks, and prioritizing preventive genetic screening are critical steps toward ensuring that SMA patients receive the full spectrum of care necessary for improving their functional outcomes and overall quality of life. Future research should focus on the identification of context-specific barriers and the evaluation of targeted interventions to alleviate these persistent challenges.

## Data Availability

The data that support the findings of this study are available from the corresponding author upon reasonable request.

## Footnotes

1 The costs convert to year 2025 based on RVU.

## Declarations

### Transparency statement

Bita Montazeri affirms that this manuscript is an honest, accurate, and transparent account of the study being reported; that no important aspects of the study have been omitted; and that any discrepancies from the study as planned (and, if relevant, registered) have been explained.
